# Anesthetic management of women with primary immunodeficiencies in the obstetric setting: A French cohort study (ANEU-DIP)

**DOI:** 10.1016/j.jacig.2025.100433

**Published:** 2025-01-30

**Authors:** Aude Beloeuvre, Olivia Anselem, Asmaa Tazi, Hawa Keita-Meyer, Nizar Mahlaoui, Caroline Charlier

**Affiliations:** a Infectious Diseases Unit, Paris Centre University Hospital, Paris, France; b Department of Obstetrics, Paris Centre University Hospital, Paris, France; c Bacteriology Department, National Reference Center for Streptococci, Paris Centre University Hospital, Paris, France; f Obstetric and Pediatric Anesthesiology Department, Assistance Publique-Hôpitaux de Paris (AP-HP), Paris Centre University Hospital, Paris, France; d Institut Cochin, Team Bacteria and Perinatality, Université Paris Cité, Inserm U1016, CNRS UMR8104, Paris, France; e University Hospital Federation Prem'impact, Paris, France; g Unité de Recherche EA 7323 Pharmacologie et Évaluation des Thérapeutiques Chez l’Enfant et la Femme Enceinte, Université de Paris Cité, Paris, France; h French National Reference Center for Primary Immune Deficiencies, Necker-Enfants malades University Hospital, AP-HP, Paris, France; i Pediatric Immuno-Hematology and Rheumatology Unit, Necker-Enfants malades University Hospital, AP-HP, Paris, France; j French National Reference Center and WHO Collaborating Center *Listeria*, Paris, France; k Biology of Infection Unit, Inserm U1117, Institut Pasteur, Paris, France

**Keywords:** Primary immune deficiency, inborn errors of immunity, registry, pregnancy, outcome, neonate, anesthesia, rare diseases, chronic diseases, infection, intervention

## Abstract

**Background:**

Primary immunodeficiencies (PIDs) encompass a large group of inherited diseases affecting the immune system. PID management is improving, enabling more patients to carry a pregnancy to term. Anesthetic care of those patients, especially obstetric neuraxial anesthesia and the associated infectious complications, has never been evaluated in this population.

**Objective:**

This retrospective multicenter study aimed to assess the anesthetic management of women with PIDs during childbirth, focusing on potential infectious complications related to neuraxial anesthesia.

**Methods:**

The medical records of 30 women aged 18 years or older, who are included in the French national PID registry (Reference Centre for Primary Immunodeficiencies [CEREDIH]) and who gave birth at one of the Assistance Publique-Hôpitaux de Paris maternity units between 2014 and 2024, were analyzed. Data on PID history, obstetric outcomes, and peripartum anesthesia were collected (the ANEU-DIP study, ClinicalTrials.gov identifier NCT06449066).

**Results:**

We examined 51 deliveries (including 20 cesarean sections) among 30 women with PIDs (13 with predominantly antibody defects, 11 with T-cell immune deficiencies, and 6 with innate immune deficiencies). Of the 49 locoregional anesthesia procedures performed, 36 were epidurals, 8 were spinals, and 5 were combined spinal-epidurals. No anesthesia-related complications were reported. The distribution and severity of PIDs in the cohort were consistent with those in other French studies. Three intrauterine infections were identified, of which 2 were associated with known risk factors and subsequent favorable maternal and neonatal outcomes.

**Conclusion:**

This study highlights the frequent use of neuraxial anesthesia in women with PIDs. No anesthesia-related complications were observed. Further research is needed to implement tailored anesthesia guidelines for this vulnerable segment of the pregnant population.

## Introduction

Primary immunodeficiencies (PIDs) encompass a large group of inherited disorders affecting the immune system, leading to susceptibility to infections and immunopathologic manifestations, including inflammation, autoimmunity, allergy, lymphoproliferation, and malignancies. Despite their prevalence being consistently underestimated, there has been a notable increase in diagnosed cases over the past decade.^1^ Advancements in the management of patients have led to significantly improved life expectancy and quality of life, enabling patients to carry pregnancies to term. This brings new challenges regarding infection risks during labor and delivery, such as endometritis, skin and soft tissue infections, urinary tract infections, and surgical site infections. To address these challenges, obstetric and anesthetic care include preventive, prophylactic, and evidence-based empiric interventions. Although pregnancies in patients with PID might be associated with higher risk of infectious complications,^2^ data to support specific anesthetic protocols are still lacking.

From this perspective, regional anesthesia requires urgent assessment. Epidural analgesia is indeed the leading neuraxial analgesia technique procedure during labor. It is administered to 82% of French women^3^; regional anesthesia (spinal or epidural) is also the preferred technique during caesarean deliveries. These approaches are associated with infectious complications that encompass (1) urinary tract and skin and soft tissue infections; (2) epidural abscesses, which complicate 1 in 145,000 epidural analgesia procedures,^4^ mainly in immunocompromised patients with prolonged catheter use; and (3) nosocomial meningitis related to aseptic meningeal breaches after spinal anesthesia (occurring in 1.1 in 100,000 procedures), mainly in healthy patients.^4^ The extent and management of these complications have been addressed in patients with acquired immunodeficiencies but not in women with PID. Here we have aimed at filling this gap by studying the anesthetic management of patients with PID in the obstetric setting, focusing on anesthetic complications, including infectious ones.

## Results and discussion

ANEU-DIP was a retrospective multicenter study. Eligible patients were women aged 18 years or older who were included in the French registry Reference Centre for Primary Immunodeficiencies (CEREDIH) and gave birth in an Assistance Publique-Hôpitaux de Paris (APHP) maternity unit between 2014 and 2024. The APHP regroups 39 tertiary and teaching university hospitals in the Greater Paris area (12 million inhabitants).

PIDs were classified according to the International Union of the Immunological Societies 2022 classification. Infections were categorized as severe if they involved invasive bacterial, fungal, or viral pathogens requiring intensive care or surgery; other infections were considered mild. Preterm birth was defined as birth before 37 weeks of gestation (WG) and categorized as severe (occurring at 24-31 WG) or moderate or late (occurring thereafter). Prematurity was identified as spontaneous or medically indicated if labor was induced for medical reasons. Intrauterine infection and endometritis were defined according to National French College of OBGYN guidelines.^5^^,^^6^

Data were obtained from medical records, encompassing epidemiologic information, PID history, obstetric outcomes, peripartum anesthesia details (type, duration, and antibiotic prophylaxis), and any local or systemic complications (including infections within 6 weeks postpartum).

Ethics committee approval was obtained on May 23, 2024 (approval no. AAA-2024-10025). In line with the French guidelines, nonopposition after written information was collected for each participant. The study adhered to the General Data Protection Regulation according to Reference Methods MR004 APHP registration (ClinicalTrials.gov identifier NCT06449066).

A total of 409 women from the Reference Centre for Primary Immunodeficiencies registry who had been born between 1973 and 2006 were screened. Of these, 57 gave birth between 2014 and 2024, including 31 who delivered in an APHP maternity unit. One woman declined participation, resulting in a final cohort of 30 patients who experienced 51 deliveries across 9 maternity units (Table I**)**. The PIDs consisted of 13 predominantly antibody innate immunity deficiencies (43%), 11 T-cell innate immunity deficiencies (37%), and 6 innate immunity deficiencies (20%). Seven deliveries occurred before PID diagnosis.

**Table I:** Characteristics of the 30 included women

Characteristic	Value
Age at delivery (y), median (IQR)	31 (29-36)
PID type, no. (%)	
Predominantly antibody deficiency∗	13 (43)
T-cell immune deficiency†	11 (37)
Innate immune deficiency‡	6 (20)
Age at PID diagnosis (y), median (IQR)	12.5 (1-28)
PID diagnosis made before the first delivery, no. (%)	23 (77)
ASA physical status score at delivery, median (IQR)	2 (2-2)
Medical history including PID, no. (%)	
Severe§/mild infection‖	11 (37); 27 (90)
Including opportunistic invasive infection∗∗	2 (7)
Neoplasia††	2 (7)
Autoimmunity‡‡	9 (30)
PID treatment before pregnancy onset, no. (%)	
Hematopoietic stem cell transplantation§§	1 (3)
IgRT	16 (53)
Immunosuppressive therapy‖‖	7 (23)
Other¶¶	3 (10)
Anti-infectious prophylaxis before pregnancy, no. (%)	
Antibacterial∗∗∗	10 (33)
Antiviral†††	5 (17)
Antifungal‡‡‡	2 (7)
Additional comorbidities, no. (%)	
Obesity defined by a body mass index > 30	1 (3)
Type I diabetes mellitus	1 (3)
History of early fetal loss, no. (%)	6 (20)

*ASA*, American Society of Anesthesiology; *IQR*, interquartile range.

∗ Common variable immune deficiency (n = 4); IgA and IgG subclass deficiency (n = 4); specific antibody deficiency (n = 2); *CARD11* gain-of-function mutation; sideroblastic anemia with immunodeficiency, fevers, and developmental delay) syndrome (*TRNT1* mutation); and trichothiodystrophy (n = 1 each).

† CTLA4 deficiency (n = 2), RLTPR mutation (n = 3), POLE 3 mutation (FILS syndrome [n = 2]), autoimmune lymphoproliferative syndrome (n = 2), Di George syndrome, and familial hemophagocytic lymphohistiocytosis syndrome (n = 1 each).

‡ Chronic granulomatous disease (n =2), chronic neutropenia, TLR3 mutation, congenital asplenia (RPSA mutation), and factor I deficiency (n = 1 each).

§ Infections were defined as severe (ie, any invasive bacterial, fungal, or viral infection, as well as any infection requiring intensive care or surgical management) and otherwise as mild. Severe infections consisted of bacterial meningitis involving *Streptococcus pneumoniae* (n = 3) or *Neisseria meningitidis* (n = 1), herpes simplex virus meningoencephalitis, spontaneous multifocal *S agalactiae* spondylodiscitis, severe influenza requiring intensive care unit management, severe *Haemophilus influenzae* pneumonia requiring intensive care unit management, and *Streptococcus pyogenes* myositis (n = 1 each).

‖ Mild infections consisted of pneumonia, bronchitis, pyelonephritis, ear nose and throat infections, and skin abscesses.

∗∗ One patient with CTLA4 deficiency presented with disseminated *Mycobacterium genavense* infection, and 1 patient with chronic granulomatous disease presented with 2 episodes of pulmonary aspergillosis.

†† Upper respiratory tract carcinoma and duodenal B-cell lymphoma (n = 1 each).

‡‡ Idiopathic thrombocytopenic purpura (n =3), Hashimoto thyroiditis (n = 2), autoimmune hemolytic anemia, autoimmune neutropenia, Crohn disease, and Evans syndrome (n = 1 each).

§§ One patient had undergone successful hematopoietic stem cell transplantation during childhood for familial hemophagocytic lymphohistiocytosis.

‖‖ The immunosuppressants used were corticosteroids (n = 2); azathioprine (n = 2); and rituximab, tocilizumab, and vedolizumab (n = 1 each).

¶¶ Other medications consisted of abatacept (n = 2) and hydroxychloroquine (n = 1).

∗∗∗ Cotrimoxazole (n = 6), azithromycin and cloxacillin (n = 3 each), and amoxicillin (n = 2).

††† Valaciclovir in all cases.

‡‡‡ Itraconazole in both cases.

In all, 11 women reported severe infection (35%), mostly bacterial meningitis, severe pneumonia, or opportunistic infection; 16 (53%) had been undergoing immunoglobulin replacement therapy (IgRT) before pregnancy, and 5 were receiving immunosuppressive treatment (16%). One had undergone hematopoietic stem cell transplantation in infancy (Table I).

Of the 51 pregnancies examined, 17 (33%) were complicated by viral, bacterial, or parasitic infections, with hospital admission required in 2 of them. Anti-infective prophylaxis was administered in 22 pregnancies (in 22 of 51 [43%]). One pregnancy was medically terminated at 20 WG for a non–PID-related event. All of the others led to a live birth. A total of 20 deliveries were via caesarean section (20 of 51 [39%]), including 10 on an emergency basis (Table II^7^).

**Table II:** Pregnancy and delivery characteristics

Characteristic	Value (N = 51)
Pregnancy	
Gestatity at last delivery (no.), median (IQR)	2 (1-4)
Parity before last delivery (no.), median (IQR)	1 (0-2)
Infertility treatment, no. (%)	0
Twin pregnancies, no. (%)	2/51 (4%)
Pregnancy complications, no. (%)	
Obstetric complications, no. (%)∗	11/51 (21%)
Infectious complications, no. (%)†	12/51 (23%)
Other complications, no. (%)‡	2/51 (4%)
Time between PID diagnosis and delivery (y), median (IQR)	28 (10-33)
Anti-infectious prophylaxis during pregnancy, no. (%)	20/51 (39%)
Antibacterial, no. (%)§	9/51 (18%)
Antiviral, no. (%)‖	6/51 (12%)
Antifungal, no. (%)¶	7/51 (14%)
IgRT during pregnancy, no. (%)∗∗	28/51 (55%)
Immunosuppressive therapy during pregnancy, no. (%)††	5/51 (10%)
Delivery	
Labor duration (h), median (IQR)	7.6 (4-8)
Premature labor, no. (%)	6/51 (12%)
Moderate, no. (%)‡‡	6/6 (100%)
Medically induced, no. (%)§§	2/6 (33%)
Induced labor, no. (%)‖‖	18/42 (43%)
Medical indication, no. (%)¶¶	16/18 (89%)
Caesarean section, no. (%)	20/51 (39%)
Emergency caesarean section∗∗∗	10/20 (50%)
Planned caesarean section†††	10/20 (50%)
Vaginal delivery analgesia and anesthesia	
Epidural, no. (%) | epidural catheter duration (h) median (IQR)	27/31 (87%) | 7 (3-9)
Spinal, no. (%)‡‡‡	3/31 (10%)
Caesarean section anesthesia, no. (%)	
Epidural	9/20 (45%)
Spinal	5/20 (25%)
Combined spinal epidural§§§	5/20 (25%)
General‖‖‖	1/20 (5%)
Indication for antibiotic prophylaxis during labor and delivery, no. (%)	32/51 (63%)
Prevention of neonatal *S agalactiae* infection	
Prolonged rupture of membranes	5/5 (100%)
Uterine revision	7/7 (100%)
Cesarean section	5/5 (100%)
Indication not specified¶¶¶	20/20 (100%)
Antibiotic therapy during labor and delivery, no. (%)	2/32 (6%)
Maternal fever, no. (%)∗∗∗∗	10/51 (20%)
Intrauterine infection, no. (%)††††	7/10 (70%)
Complications, no. (%)	3/10 (30%)
Anesthesia and analgesia related complications	0/51 (0%)
Infectious complications‡‡‡‡	8/51 (14%)
Endometritis	2/51 (4%)
Surgical site infection	2/51 (4%)
Bacteriemia	1/51 (2%)
Pyelonephritis, no. (%)	1/51 (2%)
Obstetric complications, no. (%)§§§§	4/51 (8%)
Postpartum hemorrhage‖‖‖‖	3/51 (6%)

*HELLP*, Hemolysis, elevated liver enzymes low platelet syndrome; *IQR*, interquartile range.

∗ Gestational diabetes (n = 5), including 2 pregnancies requiring insulin, pregnancy cholestasis (n = 2), thrombopenia (n = 3) and severe preeclampsia complicated with HELLP syndrome (n = 1).

† Bronchitis (n = 3); pyelonephritis (n = 3); bacterial pneumonia (n = 2), with both cases requiring hospitalization; recurring urinary infections (n = 2); cytomegalovirus reactivation (n = 2); cutaneous abscess (n = 1); herpes zoster (n = 1); and taenia (n = 1).

‡ Two patients with chronic epilepsy presented with generalized tonic-clonic seizures.

§ Oxacillin (n = 4), azithromycin (n = 3), cotrimoxazole (n = 1), and amoxicillin (n = 1).

‖ Valaciclovir in all cases.

¶ Itraconazole in all cases.

∗∗ One patient received IgRT during pregnancy only.

†† The immunosuppressants used were azathioprine (n = 2), vedolizumab (n = 2), and corticosteroids (n = 1).

‡‡ Moderate prematurity was defined as preterm birth within 32 WG and 36 plus 6 WG.

§§ Premature labor was induced for intrauterine growth retardation and severe preeclampsia with HELLP syndrome (n = 1 each).

‖‖ Planned cesarean sections were not included in this count.

¶¶ Labor was induced for postterm pregnancy (n = 4), macrosomia (n = 2), and intrauterine growth retardation (n = 2), as well as for fetal heart rhythm anomalies, suspicion of intrauterine infection, thrombopenia, therapeutic anticoagulation, membrane rupture without spontaneous labor, gestational diabetes, the mother’s general altered condition, and medical termination of pregnancy (n = 1 each).

∗∗∗ Performed for fetal heart rate anomalies (n = 4), failure of labor induction (n = 2), nonengagement at full dilatation (n = 2), HELLP syndrome, and suspected intrauterine infection with labor dystocia (n = 1 each).

††† The undications were previous cesarean section (n = 8), previous perineal tear, and severe transit disorder (n = 1 each).

‡‡‡ Spinal anesthesia was performed for uterine revision in all 3 cases.

§§§ The indication for combined spinal-epidural anesthesia was multiple previous cesarean sections.^7^

‖‖‖ General anesthesia was performed because of concomitant severe thrombopenia.

¶¶¶ Two patients received antibiotic prophylaxis without any specified indication, amoxicillin and cefotaxime (n = 1 each).

∗∗∗∗ Amoxicillin (n = 4), amoxicillin/clavulanate (n = 2), and clindamycin (n = 1). One patient developed a fever but did not receive antibiotic therapy.

†††† Amoxicillin associated with gentamicin, cefotaxime associated with gentamicin, and imipenem (n = 1 each).

‡‡‡‡ Additionally, 2 patients exhibited hyperthermia postpartum and were administered antibiotic therapy.

§§§§ One patient presented with wall hematoma requiring surgery.

‖‖‖‖ One patient required blood transfusion.

Among the 31 patients who underwent vaginal deliveries, 27 (87%) chose epidural analgesia, 3 (10%) required spinal anesthesia, and 1 chose to give birth without analgesia. All scheduled caesarean sections were performed under spinal or combined spinal-epidural anesthesia. Patients with an unscheduled caesarian section received either spinal or epidural anesthesia, except for 1 with hemolysis, elevated liver enzymes, and low platelet count syndrome and severe thrombopenia, who required general anesthesia. Locoregional anesthesia required multidisciplinary assessment in 9 cases (9 of 51 [18%]), but PID was never the reason to contraindicate locoregional anesthesia. Antibiotic prophylaxis was implemented in accordance with French guidelines in all cases: caesarian sections benefited from antibiotic prophylaxis (cefazoline or clindamycin in all 20 cases [100%]); penicillin-based prophylaxis was used in 5 cases of *Streptococcus agalactiae* vaginal colonization and during each uterine revision (n = 5).^8^

No anesthesia-related complications, infectious or otherwise, were reported. No patient required readmission to the hospital after maternity discharge. Among the patients who returned for the 6-week postpartum consultation (32 of 51 [63%]), none reported anesthesia-related complications.

Three patients presented with postpartum hemorrhage (6%), with 1 requiring blood transfusion. One patient developed severe preeclampsia and hemolysis, elevated liver enzymes, and low platelet count (HELLP) syndrome, which improved rapidly following delivery.

Of the 51 deliveries, 8 (16%) were complicated by fever during labor. In 3 cases, intrauterine infection was identified: 2 cases in patients with a T-cell deficiency (*RLTPR* deficiency under IgRT and azithromycin and *POLE3* mutation, also under IgRT) and 1 case in a patient with familial hemophagocytic lymphohistiocytosis who had undergone successful hematopoietic stem cell transplantation in infancy (having remained free from severe infections thereafter, she did not receive any prophylaxis). Documentation involved *Escherichia coli*, *S agalactiae,* and *Gardnerella vaginalis* (n = 1 case each). In 2 of these 3 cases, risk factors for intrauterine infection (ie, prolonged membrane rupture and prolonged labor) were in evidence. Two of these patients (1 who underwent an emergency caesarian section and 1 who experienced prolonged labor) subsequently developed endometritis, including 1 with concurrent *Bacteroides fragilis* bacteremia (Table II).

Of the 20 patients, 2 (10%) experienced surgical site infections after emergency caesarian sections, with 1 of them requiring surgery. One patient developed postpartum *E coli* pyelonephritis. Additionally, 2 patients exhibited postpartum hyperthermia and received amoxicillin-clavulanate until infection could be ruled out.

We examined 49 neuraxial anesthesia procedures performed on 30 patients with PIDs during labor. Importantly, we did not find evidence of any anesthesia-related complication, including no neurologic infection. The study’s main strength lies in its representativeness; median age aligned with the typical childbearing age in France, and the distribution and severity of PIDs matched those reported in French studies.^9^

The study’s limited sample size mirrored the low incidence of PIDs and hampered a precise assessment of the most dreadful consequences of neuraxial anesthesia, namely, neurologic infections, which encompass postepidural abscesses (the incidence of which is estimated at around 1 in 145,000 for all indications^10^) and infectious meningitis following spinal anesthesia (the incidence of which is around 1.1 cases per 100,000 procedures), predominantly reported in an obstetric context.^11^

Data about these complications in immunocompromised individuals remain scarce. No complication was reported in a 1997 retrospective study evaluating neuraxial anesthesia performed during labor in 36 American HIV-infected women (all of whom were receiving antiretroviral therapy and none of whom was at the AIDS stage).^12^ Similarly, studies involving children undergoing kidney transplantation (n = 50) and receiving perioperative or postoperative epidural analgesia or adults with liver transplantation (n = 67) and receiving thoracic epidural analgesia) did not report any infectious complication associated with these procedures.^13^^,^^14^

Published data on patients with PID in the obstetric setting are almost inexistant, as they are limited to 3 case reports: a case involving a patient with common variable immunodeficiency who developed aseptic meningitis following spinal anesthesia,^15^ a case involving a pregnant patient with hyper-IgE syndrome who developed *S aureus* hip arthritis and did not receive an epidural during labor to avoid local seeding,^16^ and a case involving a patient with selective IgG3 deficiency who developed an epidural abscess following thoracic epidural catheter placement for pain management.^17^

These observations underscore the need for specific anesthetic care for patients with PIDs. Here, PIDs were never considered a contraindication to neuraxial anesthesia, even in women with a history of severe infections such as meningitis or spondylodiscitis. The frequent use of neuraxial anesthesia, with only 2 deliveries occurring without it, suggests a possibly more medicalized approach to pregnancy management in these patients, although bias related to the sample size and local protocols could not be ruled out. The elevated rates of cesarean sections and labor inductions mirrored those reported in transplant recipients and likely reflect both the underlying health conditions and the increased medical surveillance that these patients receive.^18^

Although fever during labor was uncommon, 3 cases of intrauterine infections were reported, with 2 of them in the context of concomitant identified risk factors.^19^ All involved typical pathogens^19^ with good outcome and no recurrence in subsequent pregnancies.

The retrospective design of this study introduces biases, particularly in assessing the incidence of other infections potentially related to epidural anesthesia, such as urinary tract infections. These limitations highlight the need for further research to better evaluate the safety of neuraxial anesthesia in patients with PIDs and to develop tailored guidelines for managing this unique and vulnerable population.

**Clinical implication**

Use of neuraxial anesthesia for delivery in women with PIDs appears safe. No anesthesia-related complications were observed.
